# Women’s Hospital Medical College

**Published:** 1874-03

**Authors:** 


WOMEN'S HOSPITAL MEDICAL COLLEGE. — The Commencement exercises of the Women's Hospital Medical College took place on the evening of February 24, at the First M. E. Church. The exercises were opened with music on the organ by Prof. L. Falk, followed with prayer by Rev. Mr. Chamberlain. After a vocal selection by the choir, Prof. Byford addressed the audience, as follows:

"*Ladies and Gentlemen*:— Fifteen or twenty years ago, but few women studied medicine. The few who did found it almost impossible to gain admittance into any medical school, and were sneered at by friends and the world. Now, we have ten or twelve colleges that admit them to their halls, and give them all the advantages granted to the male sex. I am glad to see the women taking advantage of these opportunities. There are now about one hundred and twenty students of this sex in the United States. Public prejudice is yielding, and they are being acknowledged as equal to the brothers of the profession. (The six members of the graduating class were called forward.) It gives me pleasure, ladies, to bestow the Degree of Medicine upon you, because it opens up a field heretofore closed to you; and it gives me pleasure, because I think you will be an honor to the profession you have chosen."

He then presented them with their diplomas.

Mrs. Carr, of the graduating class, delivered a short valedictory, speaking in honor of the faculty, and regretting that the many pleasant days they had spent together had drawn to a close. Music by Prof. Falk. Dr. Earle then addressed the graduates in behalf of the faculty. His address consisted of a review of anatomy, physiology, and words of encouragement to the graduates. Rev. Mr. Chamberlain then addressed the audience regarding the appropriateness of women entering the medical profession; and pronounced the benediction. Two of the graduates go to China as medical missionaries; some of them are now the wives of physicians; others entered to make for themselves a name in their chosen profession, alone and unaided.

